# Supplementary material for: Innate heuristics and fast learning support escape route selection in mice
Source: Curr Biol. 2022 Jul 11;32(13):2980–2987.e5. doi: 10.1016/j.cub.2022.05.020 (PMC9616796; doi:10.1016/j.cub.2022.05.020)
Supplement: Document S1. Figures S1–S3 and Table S1 [file mmc1.pdf]

Current Biology, Volume *32*

Supplemental Information

Innate heuristics and fast learning support escape route selection in mice

Federico Claudi, Dario Campagner, and Tiago Branco

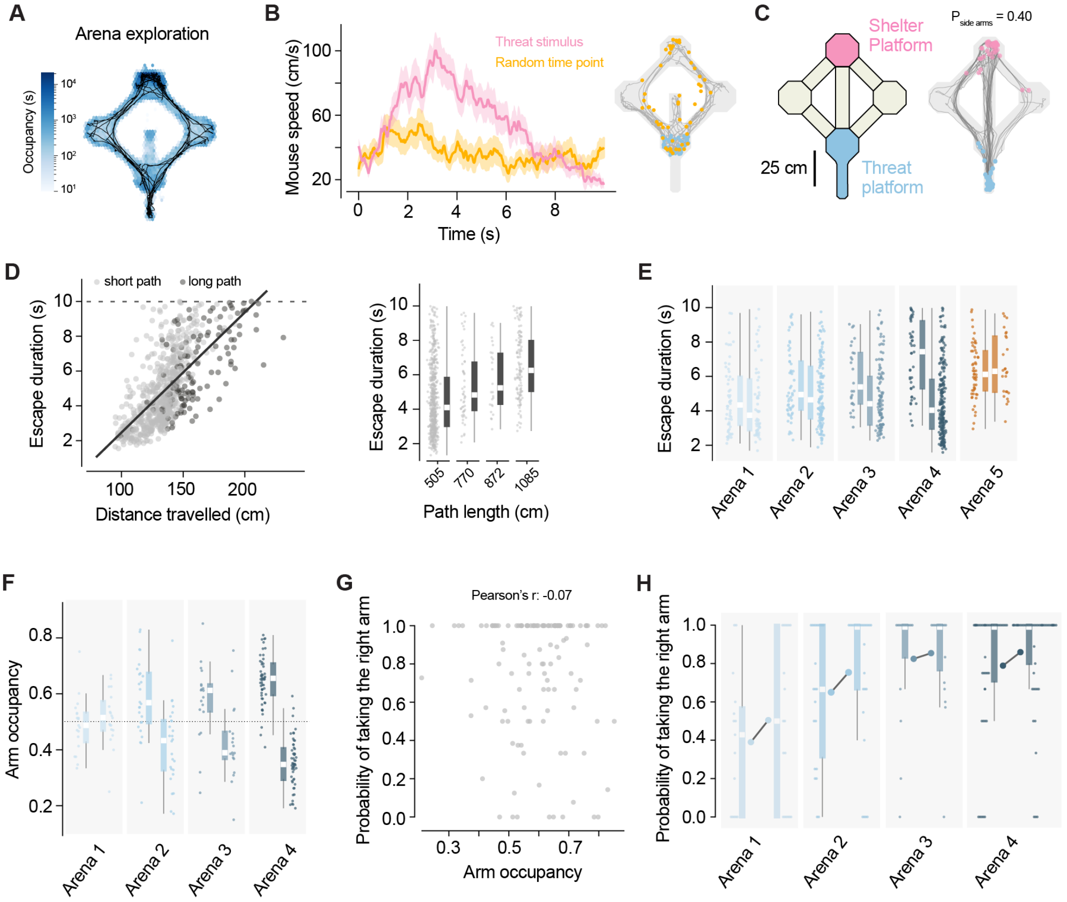

Figure S1 - Escape duration correlates with distance, and route choice does not depend on path exploration, Related to Figure 1.

(A) Heatmap of arena occupancy during exploration of Arena 1 with example tracking traces from one mouse (black). **(B)** Left, speed trajectory in the 10s following the threat stimulus (pink) or a randomly selected time point when the mouse was in the threat area (yellow). Probability of reaching the shelter after threat stimulus = 0.65 vs 0.17 for random time points (p = 0.00097, Fisher exact test). Bold curves and shaded areas show mean and standard deviation across trials. Right, tracking traces for the random time point condition, blue circles show the starting location, orange circles the final location. **(C)** Left, schematic view of a version of Arena 1 with the central arm open. Right, movement tracking traces for all escape trials (grey) for Arena 1 with the central arm open (c.f. Figure 1A). Blue and pink circles mark the start and end of each escape run, respectively. **(D)** Left, correlation between distance travelled during escape and escape duration. Each dot represents a single escape trial, darker dots are trials in which the animals selected the longer paths (in Arenas 2,3,4). Black line shows best fit of linear regression (slope: 0.07, intercept: -4.4). Right, escape duration along paths of different lengths. Each dot represents a single trial, box plots show the median and 95th percentile of the distribution. **(E)** Escape duration for left and right path escapes across arenas (for each arena the left bar shows escapes on the left and the right bar shows escapes on the right). Each dot represents a single trial, box plots show the median and 95th percentile of the distribution (t-test with Bonferroni correction for comparison between left and right: Arena 1, p=1.0; Arena 2, p=0.5; Arena 3, p=0.04; Arena 4, p=1.9e-12; Arena 5, p=1.0). **(F)** Fraction of time spent on left and right path during exploration normalized by the total time spent exploring (shown by bars on the left and right for each arena, respectively; dashed line marks equal occupancy for both arms). Circles indicate individual mice, box plots show the median and 95th percentile of the distribution (t-test with Bonferroni correction for comparison between left and right: Arena 1, p=1.0; Arena 2, p=0.0003; Arena 3, p=0.0066; Arena 4, p=8.2e-32;). **(G)** Plot showing that arm occupancy (calculated as in panel F) and probability of escaping through the right arm are not correlated. **(H)** Probability of escape on the right path for subsets of trials in which mice reached the threat platform from the left or the right path (shown by bars on the left and right for each arena, respectively). Colored circles represent individual mice, box plots show the median and 95th percentile of the distribution (values between left and right are not significantly different for all arenas).

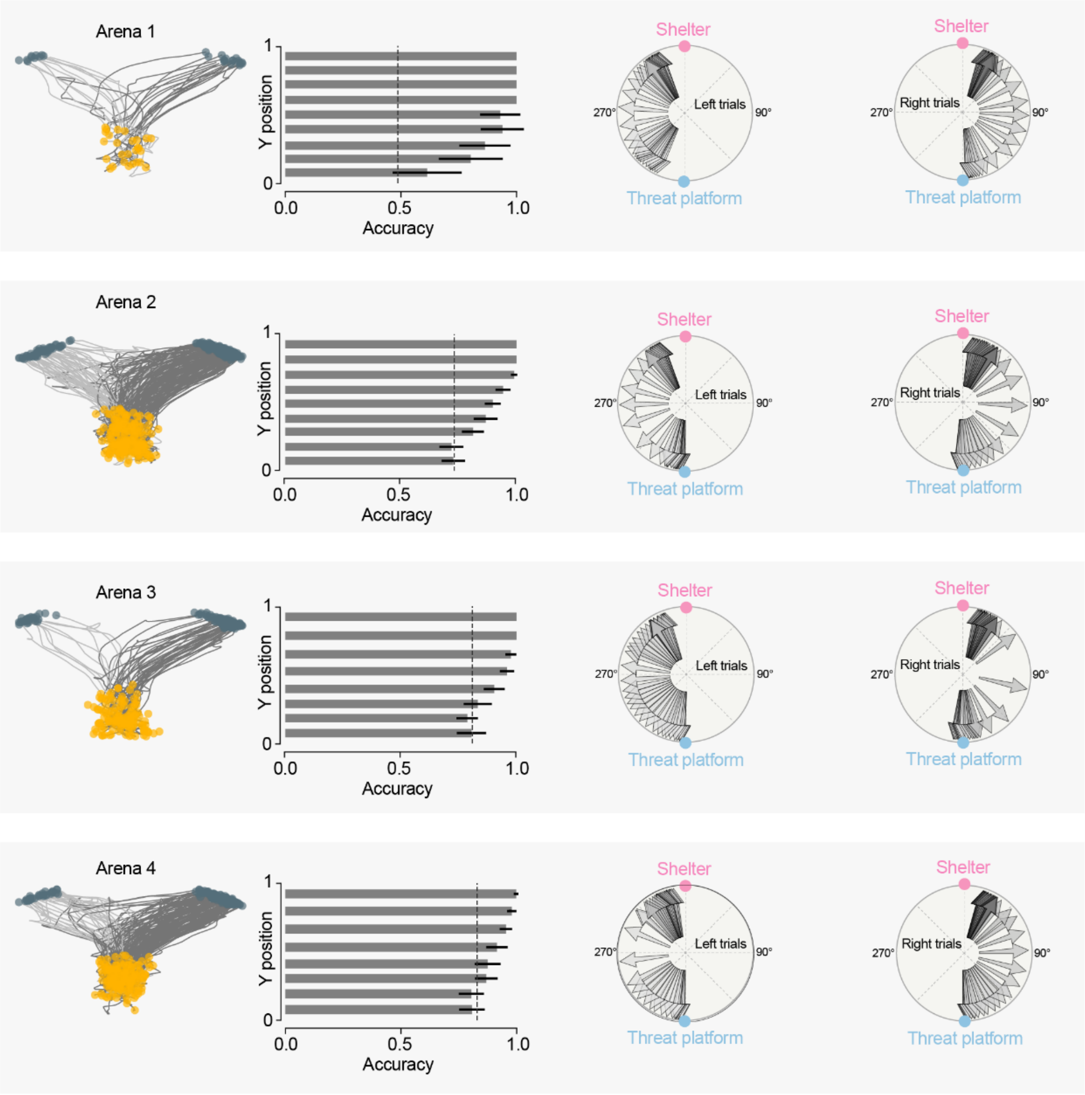

Figure S2 - Escape path choice can be decoded from the movement trajectory in the threat platform, Related to Figure 1.

Decoding of escape path selection probability based on tracking data on the threat platform. Left, tracking traces for all trials in each arena. Darker traces show escapes on the right path, orange circles the initial position and blue circles the animal's position at the time of leaving the threat platform. Center, test set accuracy of a logistic model predicting escape path choice based on tracking data binned along the threat-shelter axis. Right, average orientation across trials during escape progression for left and right trials. Arrows show orientation for each time bin, darker colors indicated later time points.

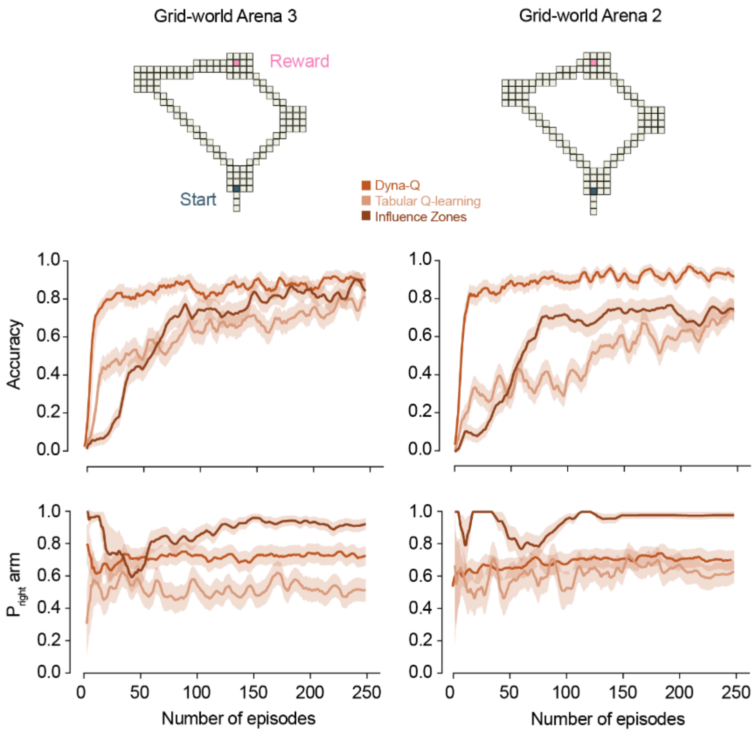

Figure S3 - Model performance in Arenas 2 and 3, Related to Figure 3.

Top row, schematics of the grid world arenas used for simulating Arenas 2 and 3. Middle row, evolution of accuracy during learning for the three different RL models under ε-greedy exploration. Bottom row, probability of choosing the right arm in successful trials during training. Note that for Dyna-Q, the probability of choosing the right arm in Arena 2 is slightly lower than in Arena 3, and both probabilities are lower than for Arena 4 (c.f. Figure 3D), which qualitatively matches the experimental data.

	DISCOUNT	LEARNING RATE	EXPLORATION RATE	EXPLORATION RATE DECAY
**QTABLE**	0.8	0.9	0.4	0.995
**DYNA-Q**	0.8	0.8	0.4	0.995
**INFLUENCE ZONES**	0.8	0.2	0.4	0.995

Table S1 – Parameters for the reinforcement learning agents, Related to Figure 3.

Parameter values for the reinforcement learning agents.
