# Supplementary material for: Innate heuristics and fast learning support escape route selection in mice
Source: Curr Biol. 2022 Jul 11;32(13):2980–2987.e5. doi: 10.1016/j.cub.2022.05.020 (PMC9616796; doi:10.1016/j.cub.2022.05.020)
Supplement: Document S2. Article plus supplemental information [file mmc6.pdf]

Current Biology

Report

Innate heuristics and fast learning support escape route selection in mice

Highlights

- Mice learn to escape via the fastest route after ~10 minutes in a new environment
- Escape routes are learned during exploration and do not require threat exposure
- Mice prefer escape routes that minimize path distance and angle to shelter
- Fast route learning can be replicated by model-based reinforcement learning agents

Authors

Federico Claudi, Dario Campagner, Tiago Branco

Correspondence

t.branco@ucl.ac.uk

In brief

Claudi et al. show that mice combine fast spatial learning with innate rules to quickly learn the fastest escape route to shelter when there are multiple possible paths.

Claudi et al., 2022, Current Biology *32*, 2980–2987
July 11, 2022 © 2022 The Authors. Published by Elsevier Inc.
https://doi.org/10.1016/j.cub.2022.05.020

CellPress
OPEN ACCESS

Current Biology

Report

Innate heuristics and fast learning support escape route selection in mice

Federico Claudi,[1] Dario Campagner,[1,2] and Tiago Branco[1,3,*]
[1]UCL Sainsbury Wellcome Centre for Neural Circuits and Behaviour, London W1T 4JG, UK
[2]Gatsby Unit, UCL, London W1T 4JG, UK
[3]Lead contact
*Correspondence: t.branco@ucl.ac.uk
https://doi.org/10.1016/j.cub.2022.05.020

SUMMARY

When faced with imminent danger, animals must rapidly take defensive actions to reach safety. Mice can react to threatening stimuli in ∼250 milliseconds[1] and, in simple environments, use spatial memory to quickly escape to shelter.[2,3] Natural habitats, however, often offer multiple routes to safety that animals must identify and choose from.[4] This is challenging because although rodents can learn to navigate complex mazes,[5,6] learning the value of different routes through trial and error during escape could be deadly. Here, we investigated how mice learn to choose between different escape routes. Using environments with paths to shelter of varying length and geometry, we find that mice prefer options that minimize path distance and angle relative to the shelter. This strategy is already present during the first threat encounter and after only ∼10 minutes of exploration in a novel environment, indicating that route selection does not require experience of escaping. Instead, an innate heuristic assigns survival value to each path after rapidly learning the spatial environment. This route selection process is flexible and allows quick adaptation to arenas with dynamic geometries. Computational modeling shows that model-based reinforcement learning agents replicate the observed behavior in environments where the shelter location is rewarding during exploration. These results show that mice combine fast spatial learning with innate heuristics to choose escape routes with the highest survival value. The results further suggest that integrating prior knowledge acquired through evolution with knowledge learned from experience supports adaptation to changing environments and minimizes the need for trial and error when the errors are costly.

RESULTS

Escape route choice is determined by path distance and angle to shelter

To investigate escape route choice, we placed mice in elevated arenas with a threat and a shelter platform connected by runways of different configurations and lengths. Previous work has shown that in simple arenas, mice escape along a direct vector toward a memorized shelter location[2,7,8] and that they can form subgoal memories to avoid obstacles when escaping.[3] To determine whether mice learn the value of alternative routes to shelter, we first built an arena where the direct path to shelter lead to a dead end while two other open paths were available (Figure 1A). Mice explored the arena over a period of ∼10 minutes (Figure S1A), after which they were exposed to innately threatening auditory and visual stimuli[8,9] when they were on the threat platform, facing away from the shelter. Mice reliably escaped from the threat (Figure S1B) and preferred the two side paths ($P_{side\ path} = 0.82$, $P_{dead\text{-}end} = 0.18$, 61 trials from 12 mice; Figure 1A; Video S1). The average time to leave the threat platform to the side arms was 2.49±0.81 s, and mice accelerated directly toward the left of right paths from the flight start (Figures 1B and 1C). In contrast, when the central arm was open, the probability of escaping along the straight path to shelter was ∼3 times higher ($P_{center} = 0.60$, 47 trials from 8 mice; $p = 5.45e{-5}$, two proportions z test; Figure S1C). This difference in path choice between the two arena configurations shows that mice quickly learn to overcome the innate preference of escaping along the shelter direction and use knowledge of which paths lead to shelter when committing to escape trajectories.

Next, we tested escape route selection in four different arenas where the length of the left arm was progressively increased while keeping constant the initial angle between each path and the threat platform (arenas 1–4 in Figure 1D; Table 1). In this experiment, the relative value of the left-hand-side path decreases between arenas 1 and 4, as mice should, in principle, escape along the shortest path to minimize exposure to danger.[10–12] The differences in geodesic distance (path length) between the threat and shelter platforms translated into differences in distance traveled and into time taken to traverse each path during escape (Figures S1D and S1E). While path choice was probabilistic, mice preferentially escaped via the shortest path in each of the three asymmetric arenas (overall $P_{right\ path} = 0.79$, 600 trials from 83 mice; Figures 1D and 1E; Video S2). The probability of taking the shortest path depended significantly on the geodesic distance ratio between the two paths (arena 1: $P_{right\ path} = 0.486$, arena 2: $P_{right\ path} = 0.731$, arena 3: $P_{right\ path} = 0.807$,

 © 2022 The Authors. Published by Elsevier Inc.
This is an open access article under the CC BY license (http://creativecommons.org/licenses/by/4.0/).

CellPress
OPEN ACCESS

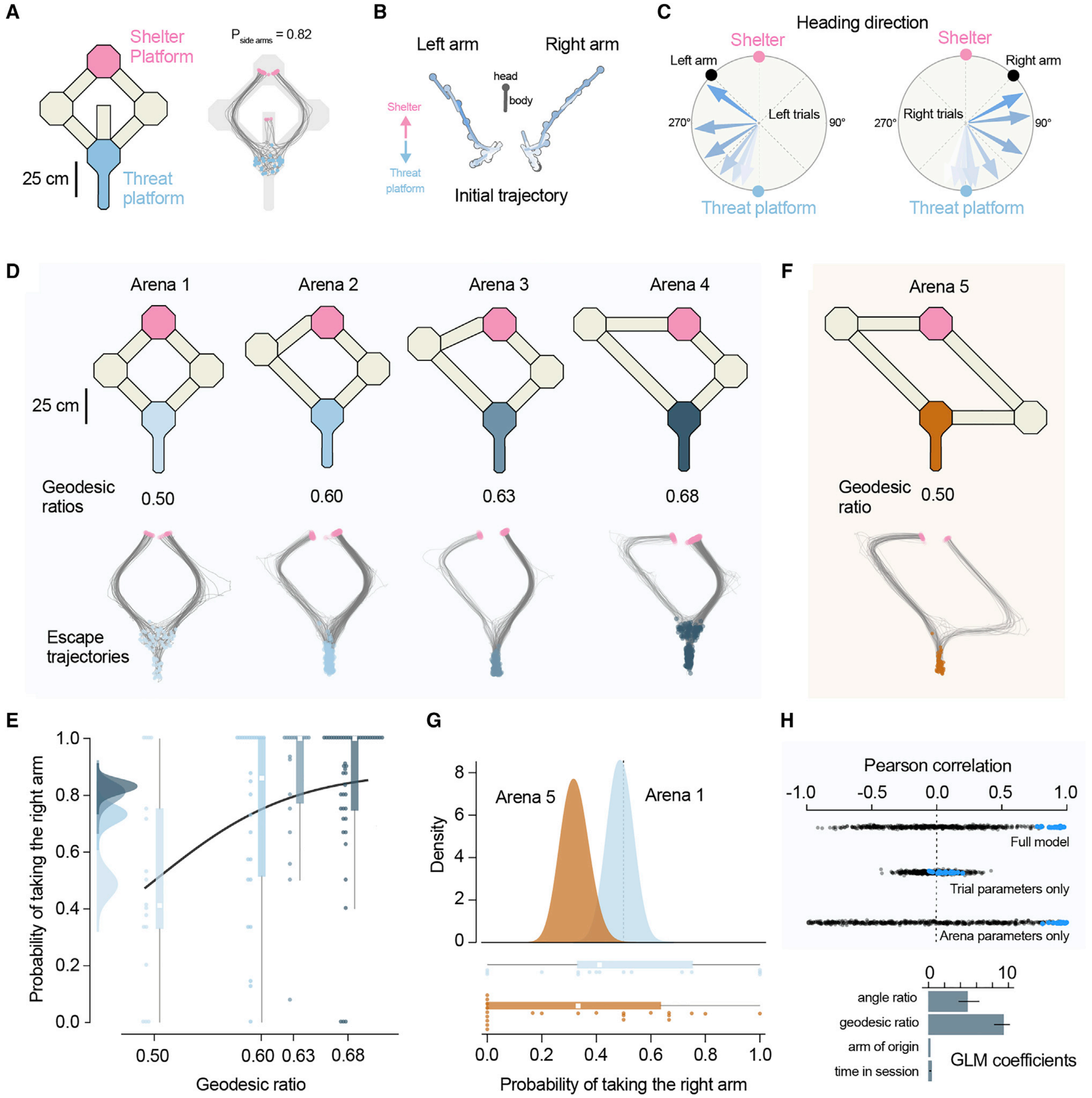

Figure 1. Escape route choice is determined by path distance and angle to shelter

(A) Left: schematic view of arena 1 with a dead-end central arm. Right: movement tracking traces for all escape trials (grey). Blue and pink circles mark the start and end of each escape run, respectively.

(B) Example trajectories on the threat platform for two escapes initiated in the left and right arm. The position of the head and body of the mouse is shown at 250 ms intervals. Color shows time elapsed from stimulus onset (later time points have darker shades of blue).

(C) Average heading direction on the threat platform for left and right escapes. Each arrow shows the average heading directions at eight time points equally spaced between stimulus onset and exiting the threat platform (pooled across trials and animals). Color shows time elapsed from stimulus onset (later time points have darker shades of blue).

(D) Top: arena schematic and corresponding geodesic ratio. Bottom: tracking traces from all trials in each arena with starting (blue) and end (pink) locations shown.

(E) Probability of escape along the right path (P_{right}) in arenas 1 to 4. Scatter dots are P_{right} of individual mice, and boxplot shows median and interquartile range for all trials pooled for each arena. The left panel shows the posterior distributions of P_{right} from the Bayesian model (STAR Methods).

(F) Top: schematic view of arena 5, with the same geodesic ratio of arena 1 but with a different angle ratio between the two arms. Bottom: tracking traces from all trials in arena 5 with start (orange) and end (pink) locations indicated.

(legend continued on next page)

Table 1. Arena properties

	Arena 1	Arena 2	Arena 3	Arena 4	Arena 5
Left path length (cm)	505	770	872	1,085	1085
Right path length (cm)	505	505	505	505	1,085
Left path angle	45°	45°	45°	45°	45°
Right path angle	45°	45°	45°	45°	90°
Geodesic ratio	0.50	0.60	0.63	0.68	0.5
Angles ratio	0.50	0.50	0.50	0.50	0.33

Lengths and angles (relative to shelter direction) of left and right paths in arenas 1–5. The geodesic and angles ratios were defined as: $ratio = \frac{left}{left + right}$.

arena 4: $P_{right\ path} = 0.829$, $p = 1.76e{-}10$, χ^2 test). The preference for the shortest path could not be explained by mice being more familiar with the short arm nor by choosing the arm they entered the threat platform from (Figures S1F–S1H). In addition, the time to leave the threat platform was independent of path chosen (2.53±1.51 and 2.58±1.37 s for left and right arm respectively, p = 0.66, t test), and path choice could be predicted from the escape trajectory before mice left the threat platform (Figure S2). This indicates that mice commit to one path from escape onset. Together, these data suggest that mice evaluate either geodesic distance or escape duration to shelter when choosing escape routes. These two quantities are strongly correlated in our experiments (Figure S1C), and we cannot disambiguate between the two alternatives. In either case, mice quickly learn to select the fastest escape routes to safety.

To assess whether other aspects of path geometry influenced escape route selection, we built an arena where the two arms had the same length but where the initial angle relative to the shelter was larger for the right arm (Figure 1F). In this configuration, escaping along the right arm initially moves the mouse away from the shelter, but the path length and escape duration is the same for both arms (Figure S1E). If mice selected escape paths based on path length or travel duration alone, they should therefore choose each path with equal probability. Instead, we found a clear preference for the left arm ($P_{left\ path} = 0.69$, 81 trials, 23 mice, p = 0.02, χ^2 test; Figure 1G). This suggests that in addition to geodesic distance, mice also consider shelter direction when choosing escape paths. To further quantify escape preference variables, we used a generalized linear model (GLM) to predict escape path choice across trials. The model could explain more than 90% of the variance in path choice in cross-validated tests and showed approximately equal weighting between geodesic distance and shelter angle, with minimal weight for exploration time and arm of origin (Figure 1H). Together, these results show that when faced with two possible paths to shelter, mice quickly learn distances and angles to shelter and escape along the route that minimizes both.

Route learning does not require escape experience and is flexible

Computing escape route choices requires at least two steps: learning the properties of the available paths to safety and a function to map those properties into their value for escape (e.g., favoring escape along the shortest path). While the first is likely learned during natural exploration of the environment, the second could, in principle, be learned through repeated encounters with threat or be innate (i.e., the animal is born with a value function that links path properties to escape values). To distinguish between these two alternatives, we computed path choice probabilities for the first trial of threat presentation (naïve) and compared them with the probabilities for trials after experience with threats (experienced). We found that the preference for shorter paths and smaller angles to shelter was already present in naïve trials and that path choice probabilities were similar between naïve and experienced trials (Figure 2A). In addition, choice probabilities did not significantly change over the course of the experimental session and repeated threat presentations (Figure 2B). This analysis suggests that the strategy for selecting escape routes does not develop through experience of escaping. Instead, the evaluation of path length and angle to shelter represents an innate heuristic that assigns escape value to the different options. The preference for selecting the shortest path upon the first exposure to threat also implies that mice learned the relevant environment properties during natural exploration of the arena. In our experiments, mice spend on average 11.03±3.8 min exploring before threat presentation, during which they perform only 4.2±0.9 complete trips between the threat platform and the shelter (Figures 2C and 2D). Mice thus require a very small amount of exploration to learn spatial properties relevant for escape and have an innate function to assign escape value to alternative paths.

Combining rapid learning of path properties with an innate value function allows mice to select escape paths shortly after entering a novel environment. Next, we aimed to establish whether this can also support adaptive escape route selection in a dynamic environment. We built a version of arena 4 where the path lengths could be quickly flipped between left and right sides (Figure 2E; Video S3). After exploration and 2 to 3 threat presentation trials, we flipped the arena and let the animals explore the maze again (14.6±6.1 min, median; 6.05±2.77 threat platform to shelter trips). We then presented threats and found that the path preference during escape changed to reflect the new arena geometry—mice now took the left arm with a higher probability (baseline $P_{right\ path} = 0.641$, flipped $P_{right\ path} = 0.321$, p = 0.0014, Fisher's exact test; Figure 2F), while the time out of threat platform and orientation movement profiles were similar between baseline and flipped trials (2.16±1.10 and

(G) Top: posterior distribution for P_{right} computed with the Bayesian model for all trials in arenas 1 and 5. Bottom: P_{right} of individual mice (scatter dots) with median and interquartile range for pooled data.
(H) Top: cross-validated Pearson correlation between predicted P_{right} and observed choice behavior. Data shown for the full model and two partial models—trial parameters only (arm of origin and time) and arena parameters only (arm length and angle). Blue dots are fits to the data; black dots are fits to shuffled data. Bottom: coefficient weights for the four predictor variables included in the GLM (mean and standard deviation over repeated tests; STAR Methods).
See also Figures S1 and S2 and Video S1, S2, and S4.

CellPress
OPEN ACCESS

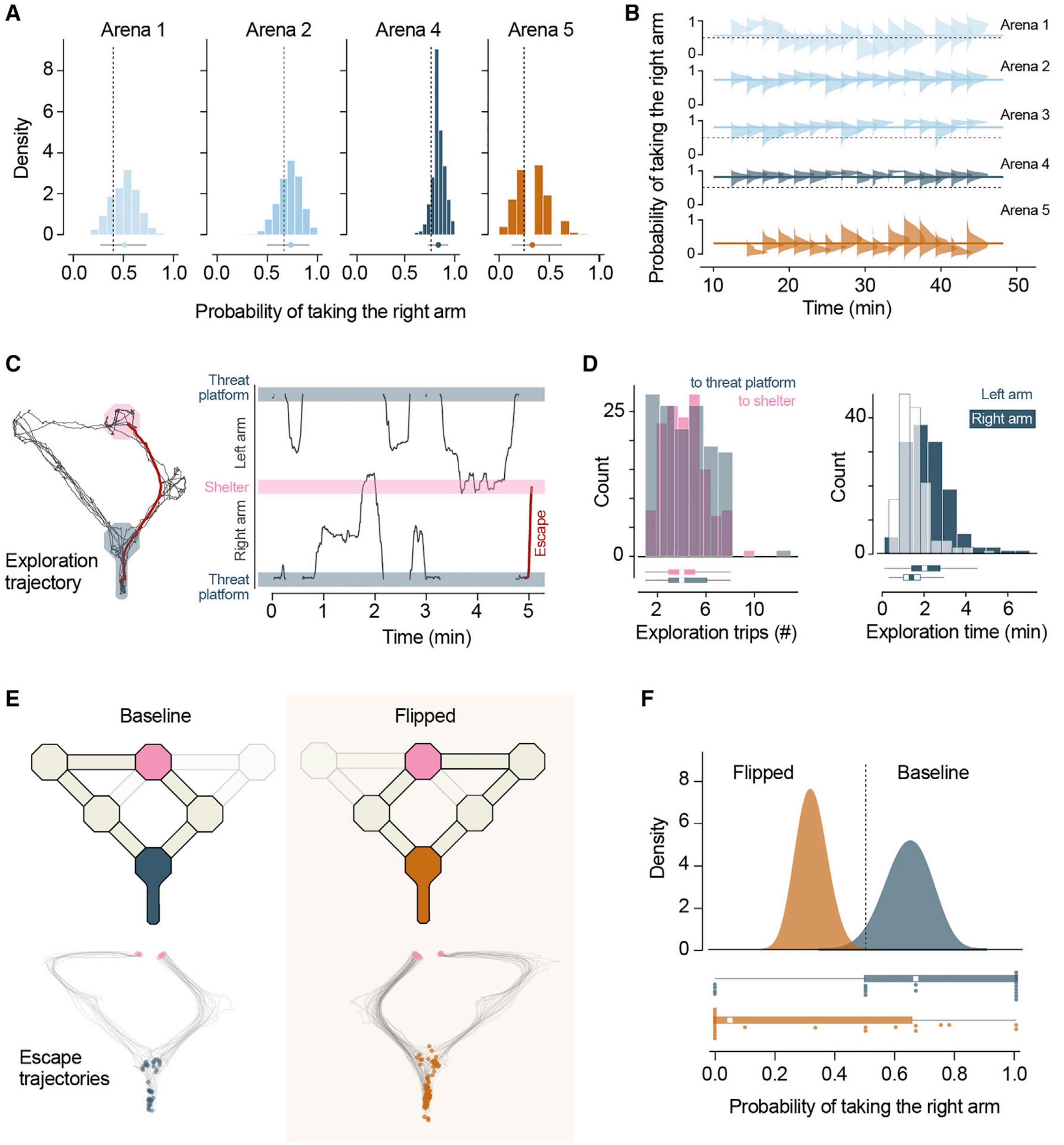

Figure 2. Route learning does not require escape experience and is flexible

(A) Distributions of P_{right} for data subsets randomly sampled from all experienced trials in each arena (mean and 95th percentile confidence shown underneath). Dashed line shows P_{right} for naïve pooled across mice.

(B) Change in P_{right} over time (within single experimental sessions). The posterior distribution of P_{right} calculated from trials binned by time in the experiment is shown for arenas 1–5 (trials pooled across animals). Solid line shows the P_{right} for the entire duration of the session.

(C) Left: example movement trajectory during arena exploration. Right: the same trajectory on the left, linearized to show the position of the mouse in the arena over time. Red trace shows the trajectory for the first escape following the exploration period.

(D) Left: histogram for the number of shelter-to-threat and threat-to-shelter trips during exploration across all experiments in arenas 1–5. Right: histogram for total time exploring the left and right arms, pooled across all arenas.

(E) Top: schematics of the dynamic arena in baseline and flipped configurations. Bottom: movement tracking trajectories for escapes in the baseline and flipped conditions (blue and orange dots show initial location; pink dots show final position).

(F) Top: Bayesian model posterior estimates of P_{right} for trials from the baseline and flipped conditions. Bottom: scatter dots show P_{right} for individual mice, and boxplot shows median and interquartile range for pooled trials.

See also Video S3.

1.77±0.93 s, respectively, p = 0.06, t test). These data suggest that after initially learning the arena geometry and developing an escape route preference, mice remain in a flexible learning state where they can incorporate new information at a rate similar to naïve animals. This ability enables the selection of the fastest escape routes in changing environments.

Model-based reinforcement agents with limited experience choose the shortest escape route

Learning the shortest escape route in our experiments was a fast process, which contrasts with the large amount of training needed for some spatial navigation and decision-making tasks,[13,14] as well as for training artificial intelligence agents.[15,16] To gain further insight into the type of learning algorithms that mice might be using, we compared the performance of different reinforcement learning (RL) algorithms[17] on a task similar to our experiments. We selected three algorithms representing different classes of RL models: Q learning (model free[17]), DYNA-Q (model based[17]) and influence zones (IZ[18]). The latter is a model-based algorithm where several state-action values are updated simultaneously according to a topological mapping between states and thus is particularly appropriate for spatial navigation tasks[18] (see STAR Methods for details on the models). These models were trained to navigate a grid-world representation of arena 4 where a positive reward was received at the shelter location (Figure 3A). As the goal of the agents is to maximize the time discounted cumulative expected reward, this should result in learning a policy that selects the shortest route to the goal, thereby mimicking innate preference of mice for selecting shorter escape paths.

We trained the RL agents under two regimes: *free-exploration*, where agents explored the environment freely under an epsilon-greedy policy for 250 episodes, and *guided-exploration*, where agents explored the environment in a single episode and moved through the maze following the exploration trajectory of individual mice recorded experimentally (STAR Methods). The *free-exploration* regime is therefore analogous to the standard practice in the RL field[17] where agents have a large number of steps to learn (up to 125,000 in our conditions). The *guided-exploration* regime poses a more challenging learning problem in principle: real exploration trajectories in our experiments have a mean of 754±267 steps, almost three orders of magnitude less (Figures 3B and 3C). Under *free-exploration*, all models learned to navigate to the goal location (Figure 3D). The short arm was chosen by 64% of the Q-learning agents that reached the shelter at the end of the test trial and by more than 95% of agents trained with DYNA-Q or IZ. (Figure 3E; see Figure S3 for performance in other arenas). In contrast, under *guided-exploration*, the Q-learning algorithm failed to learn how to reach the goal entirely (Figure 3F). The two model-based agents, however, performed significantly better, with the IZ algorithm outperforming DYNA-Q (Figure 3F). Both learned to navigate to goal for more than half of the training trajectories and chose the shorter arm for >94% of these (Figures 3E and 3F). These results suggest that rapidly learning to navigate the arena environment with limited exploration requires a learning algorithm that goes beyond naïve model-free rules and incorporates elements such as internal replay or the topology of the environment.

DISCUSSION

We have shown that mice in a novel environment learn to choose shortest escape route to shelter when there is more than one option. This learning process is fast and happens during spontaneous exploration, before mice have experienced any threats. The choice is done by selecting the route with the shortest path length and angle to shelter, which is a strategy that minimizes exposure to danger and is in line with mice keeping track of a shelter vector in open arenas.[2,7] While it may seem trivial that animals choose the fastest escape route, this need not be the case. Escape strategies in the animal kingdom are diverse, and there is often an advantage to using alternative strategies, such as outpacing the predator while not revealing where shelter is.[4,19] Perhaps surprisingly, we found that escape path choice was probabilistic. This could reflect imperfect learning of the environment geometry, noise in the sensory and decision-making systems, or the effect of unmeasured variables. Alternatively, it could provide an advantage by maintaining some exploration while exploiting the fastest known route or by increasing the unpredictability of the escape trajectory.[4,20–22]

Our results highlight a close interplay between individual experience and evolution for generating adaptive behavior. Here, mice learned the spatial properties of the arena through their natural drive and behavior but did not have to learn the value of different escape routes through experience of threat or punishment. Instead, they displayed an innate policy for escape path selection, which removes the need for trial-and-error learning in a scenario where errors could be fatal. This agrees with mice not needing to be exposed to threat to learn the direct shelter vector[2] and shelter subgoals[3] and suggests that mapping escape value onto the spatial environment is a priority of naturally behaving mice. A likely explanation is that during exploration, mice give high value to sheltering locations and routes that lead to these safe places, even in the absence of an explicit threat. They extract relevant knowledge from the environment when it is safe to do so and identify a set of possible defensive actions. When threat does come and defensive actions need to be selected, an innate heuristic is leveraged to assign value to each alternative with no need for further learning.

A key finding in this study is that route learning was fast and required minimal exploration. This builds on previous work showing fast learning of shelter location[2,8,23] and maze navigation,[11] which together suggest that since exploration of space is the natural way for mice to learn about the environment, evolution has ensured that spatial learning is fast and prioritizes supporting survival needs. It is unclear what learning algorithms mice use in the settings explored here. Our RL modeling benchmarked the performance of three different model classes, and the results suggest that a simple model-free algorithm is not sufficient to generate the observed behavior. Instead, a more sophisticated learning process, such as model-based learning, seems to be required to extract the necessary information from very limited experience. Alternatively, a simple learning algorithm could act on prior knowledge that is useful for solving the problem, such as a model for quickly estimating distances from self-motion. Additional work performing in-depth model fitting and comparison is needed to gain further insights. Another

CellPress
OPEN ACCESS

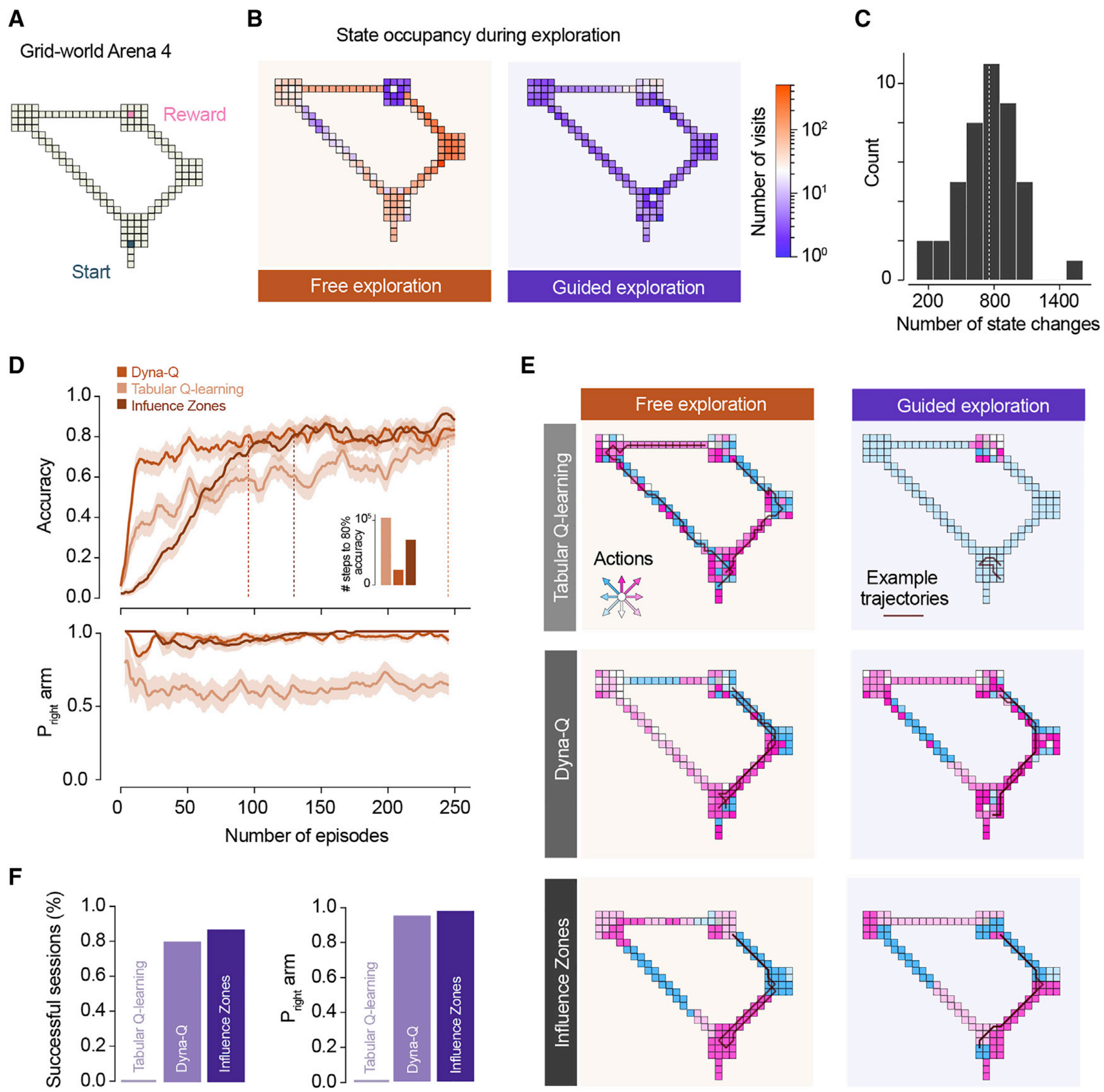

Figure 3. Model-based reinforcement agents with limited experience choose the shortest escape route

(A) Schematic of the grid world arena used for RL simulations.

(B) Heatmap for the number of visits to each state during ε-greedy (left) and guided (right) exploration (data from two representative simulations).

(C) Distribution of number of state changes during guided exploration across sessions.

(D) Learning curves for simulations under ε-greedy exploration. Top: accuracy for the different model classes tested (fraction of agents that reaches the goal state during the evaluation trial; traces are mean and standard error of the mean across multiple model instances). Dotted lines mark when 80% success rate is reached; inset shows number of training steps to reach 80% accuracy. Bottom: probability of choosing the right arm in successful trials during training for each RL model class.

(E) Illustration of the policies for the different RL simulations after training. Inset arrows show all possible actions, and the respective colors are shown in the arena to represent the best action that each class of RL models learned for every state in the arena. Lines show two example trajectories from trained agents attempting to navigate from the start to the goal location.

(F) Left: performance of agents trained under the guided exploration regime. Top: outcome (success or failure) for each class of RL algorithm across 42 sessions. Right: probability of taking the right arm in successful sessions.

See also Figure S3 and Video S3.

consideration is whether modeling the behavior as a series of state transitions is adequate. An alternative could be to simply model the decision of escaping through the left or the right path. Our choice is motivated by the observation that when exploring, mice sample the two arena arms in a piecemeal way, moving back and forth along different points in the arena (Video S4). The exploration of the two options is thus heterogeneous and often incomplete, which contrasts with traditional 1-bit choice tasks. The question remains, however, as to how many states mice abstract when performing the behavior studied here. It is interesting to note that IZ significantly reduces the state space, and it was the best performing

algorithm, perhaps indicating that the correct abstraction should have fewer states. We believe that our results invite additional work investigating the biological basis of how innate and acquired knowledge interact to generate behavior, as well as on how abstractions of this interaction can be leveraged for developing efficient learning algorithms.[24]

STAR★METHODS

Detailed methods are provided in the online version of this paper and include the following:

- KEY RESOURCES TABLE
- RESOURCE AVAILABILITY
  - Lead contact
  - Materials availability
  - Data and code availability
- EXPERIMENTAL MODEL AND SUBJECT DETAILS
  - Animals
- METHOD DETAILS
  - Behavioral arena
  - Auditory and visual stimulation
  - Behavioral assay
  - Animal tracking
  - Analysis code
  - Reinforcement learning modelling
  - Reinforcement learning models
  - Free exploration training
  - Guided exploration training
- QUANTIFICATION AND STATISTICAL ANALYSIS
  - Quantification of escape probability
  - Quantification of the probability of escaping along a dead-end
  - Quantification of heading direction
  - Quantification of arm choice probability
  - Arm choice probability for naïve vs experienced trials
  - Change in path preference with time
  - Quantification of shelter-threat trips during exploration
  - Predicting escape arm with GLM
  - Decoding escape path from threat trajectory
  - Quantification of RL models performance

SUPPLEMENTAL INFORMATION

Supplemental information can be found online at https://doi.org/10.1016/j.cub.2022.05.020.

ACKNOWLEDGMENTS

This work was funded by a Wellcome senior research fellowship (214352/Z/18/Z), a Sainsbury Wellcome Centre Core grant from the Gatsby Charitable Foundation (GAT3755 to T.B.) and Wellcome (219627/Z/19/Z to T.B.), and the Sainsbury Wellcome Centre PhD programme (to F.C.). We thank members of the Branco lab for discussions and Ruben Vale and Phillip Shamash for comments on the manuscript.

AUTHOR CONTRIBUTIONS

F.C. and T.B. designed the study and experiments and wrote the manuscript. F.C. performed all experiments and analyses. D.C. contributed to experiment design, data analysis design, and interpretation.

DECLARATION OF INTERESTS

The authors declare no competing interests.

Received: December 10, 2021
Revised: March 14, 2022
Accepted: May 9, 2022
Published: May 25, 2022

REFERENCES

1. Evans, D.A., Stempel, A.V., Vale, R., Ruehle, S., Lefler, Y., and Branco, T. (2018). A synaptic threshold mechanism for computing escape decisions. Nature *558*, 590–594. https://doi.org/10.1038/s41586-018-0244-6.
2. Vale, R., Evans, D.A., and Branco, T. (2017). Rapid Spatial Learning Controls Instinctive Defensive Behavior in Mice. Curr. Biol. *27*, 1342–1349. https://doi.org/10.1016/j.cub.2017.03.031.
3. Shamash, P., Olesen, S.F., Iordanidou, P., Campagner, D., Banerjee, N., and Branco, T. (2021). Mice learn multi-step routes by memorizing subgoal locations. Nat. Neurosci. *24*, 1270–1279. https://doi.org/10.1038/s41593-021-00884-8.
4. Cooper, W., Jr., Cooper, W.E., Jr., and Blumstein, D.T. (2015). Escaping From Predators: An Integrative View of Escape Decisions (Cambridge University Press).
5. De Camp, J.E. (1920). Relative distance as a factor in the white rat's selection of a path. Psychobiology *2*, 245–253. https://doi.org/10.1037/h0075411.
6. Snygg, D. (1935). Mazes in Which Rats Take the Longer Path to Food. J. Psychol. *1*, 153–166. https://doi.org/10.1080/00223980.1935.9917250.
7. Vale, R., Campagner, D., Iordanidou, P., Arocas, O.P., Tan, Y.L., Vanessa Stempel, A., Keshavarzi, S., Petersen, R.S., Margrie, T.W., and Branco, T. (2020). A cortico-collicular circuit for accurate orientation to shelter during escape. Preprint at bioRxiv. https://doi.org/10.1101/2020.05.26.117598.
8. Yilmaz, M., and Meister, M. (2013). Rapid innate defensive responses of mice to looming visual stimuli. Curr. Biol. *23*, 2011–2015. https://doi.org/10.1016/j.cub.2013.08.015.
9. Mongeau, R., Miller, G.A., Chiang, E., and Anderson, D.J. (2003). Neural correlates of competing fear behaviors evoked by an innately aversive stimulus. J. Neurosci. *23*, 3855–3868. https://doi.org/10.1523/jneurosci.23-09-03855.2003.
10. Ellard, C.G., and Eller, M.C. (2009). Spatial cognition in the gerbil: computing optimal escape routes from visual threats. Anim. Cogn. *12*, 333–345. https://doi.org/10.1007/s10071-008-0193-9.
11. Rosenberg, M., Zhang, T., Perona, P., and Meister, M. (2021). Mice in a labyrinth show rapid learning, sudden insight, and efficient exploration. Elife *10*, e66175. https://doi.org/10.7554/elife.66175.
12. Eason, P.K., Nason, L.D., and Alexander, J.E., Jr. (2019). Squirrels do the math: Flight trajectories in eastern gray squirrels (Sciurus carolinensis). Front. Ecol. Evol. *7*, https://doi.org/10.3389/fevo.2019.00066.
13. Tolman, E.C. (1948). Cognitive maps in rats and men. Psychol. Rev. *55*, 189–208. https://doi.org/10.1037/h0061626.
14. Aguillon-Rodriguez, V., Angelaki, D., Bayer, H., Bonacchi, N., Carandini, M., Cazettes, F., Chapuis, G., Churchland, A.K., Dan, Y., Dewitt, E., et al.; International Brain Laboratory (2021). Standardized and reproducible measurement of decision-making in mice. Elife *10*, e63711. https://doi.org/10.7554/eLife.63711.
15. Arulkumaran, K., Deisenroth, M.P., Brundage, M., and Bharath, A.A. (2017). A brief survey of deep reinforcement learning. Preprint at arXiv. https://doi.org/10.1109/MSP.2017.2743240.
16. Silver, D., Hubert, T., Schrittwieser, J., Antonoglou, I., Lai, M., Guez, A., Lanctot, M., Sifre, L., Kumaran, D., Graepel, T., et al. (2018). A general reinforcement learning algorithm that masters chess, shogi, and Go through self-play. Science *362*, 1140–1144. https://doi.org/10.1126/science.aar6404.

17. Richard, R., and Sutton, A.G.B. (2015). Reinforcement Learning - An introduction (The MIT Press).
18. Braga, A.P.d.S., and Araújo, A.F.R. (2006). Influence zones: A strategy to enhance reinforcement learning. Neurocomputing *70*, 21–34. https://doi.org/10.1016/j.neucom.2006.07.010.
19. Mattingly, W.B., and Jayne, B.C. (2005). The choice of arboreal escape paths and its consequences for the locomotor behaviour of four species of Anolis lizards. Anim. Behav. *70*, 1239–1250. https://doi.org/10.1016/j.anbehav.2005.02.013.
20. Domenici, P., Booth, D., Blagburn, J.M., and Bacon, J.P. (2008). Cockroaches keep predators guessing by using preferred escape trajectories. Curr. Biol. *18*, 1792–1796. https://doi.org/10.1016/j.cub.2008.09.062.
21. Domenici, P., Blagburn, J.M., and Bacon, J.P. (2011). Animal escapology I: theoretical issues and emerging trends in escape trajectories. J. Exp. Biol. *214*, 2463–2473. https://doi.org/10.1242/jeb.029652.
22. Domenici, P., Blagburn, J.M., and Bacon, J.P. (2011). Animal escapology II: escape trajectory case studies. J. Exp. Biol. *214*, 2474–2494. https://doi.org/10.1242/jeb.053801.
23. De Franceschi, G., Vivattanasarn, T., Saleem, A.B., and Solomon, S.G. (2016). Vision Guides Selection of Freeze or Flight Defense Strategies in Mice. Curr. Biol. *26*, 2150–2154. https://doi.org/10.1016/j.cub.2016.06.006.
24. Zador, A.M. (2019). A critique of pure learning and what artificial neural networks can learn from animal brains. Nat. Commun. *10*, 3770. https://doi.org/10.1038/s41467-019-11786-6.
25. Mathis, A., Mamidanna, P., Cury, K.M., Abe, T., Murthy, V.N., Mathis, M.W., and Bethge, M. (2018). DeepLabCut: markerless pose estimation of user-defined body parts with deep learning. Nat. Neurosci. *21*, 1281–1289. https://doi.org/10.1038/s41593-018-0209-y.
26. Yatsenko, D., Reimer, J., Ecker, A.S., Walker, E.Y., Sinz, F., Berens, P., Hoenselaar, A., James Cotton, R., Siapas, A.S., and Tolias, A.S. (2015). DataJoint: managing big scientific data using MATLAB or Python. Preprint at bioRxiv. https://doi.org/10.1101/031658.
27. Harris, C.R., Millman, K.J., van der Walt, S.J., Gommers, R., Virtanen, P., Cournapeau, D., Wieser, E., Taylor, J., Berg, S., Smith, N.J., et al. (2020). Array programming with NumPy. Nature *585*, 357–362. https://doi.org/10.1038/s41586-020-2649-2.
28. Hunter, J.D. (2007). Matplotlib: A 2D Graphics Environment. Comput. Sci. Eng. *9*, 90–95. https://doi.org/10.1109/mcse.2007.55.
29. Pedregosa, F., Varoquaux, G., Gramfort, A., Michel, V., Thirion, B., Grisel, O., Blondel, M., Prettenhofer, P., Weiss, R., Dubourg, V., et al. (2011). Scikit-learn: Machine Learning in Python. J. Mach. Learn. Res. *12*, 2825–2830.
30. Reback, J., McKinney, W., Jbrockmendel, Van den Bossche, J., Augspurger, T., Cloud, P., Gfyoung, S., Klein, A., Roeschke, M., et al. (2020). pandas-dev/pandas: Pandas 1.0.3.
31. Bradski, G., and Kaehler, A. (2000). Opencv. Dr. Dobb's Journal Software Tools 3.
32. Seabold, S., and Perktold, J. (2010). Statsmodels: Econometric and statistical modeling with python. In Proceedings of the 9th Python in Science Conference (SciPy).
33. Jockusch, J., and Ritter, H. (1999). An instantaneous topological mapping model for correlated stimuli IJCNN'99. In International Joint Conference on Neural Networks. Proceedings (Cat. No.99CH36339), *1*, pp. 529–534.

STAR★METHODS

KEY RESOURCES TABLE

REAGENT or RESOURCE	SOURCE	IDENTIFIER
Deposited data		
Deposited tracking data and metadata	Zenodo repository	https://doi.org/10.5281/zenodo.6483817
Experimental models: Organisms/strains		
Mouse: c57bl6	Charles Rivers	
Software and algorithms		
Analysis Python code	Zenodo repository	https://doi.org/10.5281/zenodo.5850541
Other		
Arena design files	This manuscript	

RESOURCE AVAILABILITY

Lead contact

Further information and requests for resources and reagents should be directed to and will be fulfilled by the lead contact, Tiago Branco (t.branco@ucl.ac.uk).

Materials availability

All tracking data and metadata, and all custom analysis code used in this work have been made freely available. All arena design files are available upon request.

Data and code availability

All data reported in this paper have been deposited at a Zenodo repository (https://doi.org/10.5281/zenodo.6483817) and is publicly available.

All original code has been deposited at a Zenodo repository (https://doi.org/10.5281/zenodo.6347161) and is publicly available.

EXPERIMENTAL MODEL AND SUBJECT DETAILS

Animals

Male adult C57BL/6J Mice (8–12 weeks old) were single housed on a 12h light cycle and tested during the light phase. Mice from the same litter were randomly assigned to different experiments when possible. Most animals (78/119) were only used once, and the remaining were used for experiments on two arenas on different days. Mice that were used twice did not show any difference in escape response or arm choice probability compared to animals that were used only once. All experiments were performed under the UK Animals (Scientific Procedures) act of 1986 (PPL 70/7652 and PFE9BCE39).

METHOD DETAILS

Behavioral arena

The behavioral arenas consisted of white acrylic platforms elevated 30cm from the floor. Each arena was composed of octagonal platforms (24cm in diameter) and connecting bridges of various lengths (10cm wide). For the experiment shown in Figure 2E, some bridge sections were fitted with a computer-controlled servo motor which rotated a 20cm long bridge section by 90 degrees in the downward direction and created a gap that the mice could not traverse. The servo motors were controlled with custom Arduino code and activated manually. The arenas were characterized by a geodesic ratio and an angles ratio (ratios of path lengths and initial segment angles, respectively). In both cases the ratios were given by $left/(left+right)$ where left/right represented the path length and angle for the two ratios respectively. See Table 1 for details of each arena.

Auditory and visual stimulation

Mice were presented with auditory stimuli consisting of three frequency modulated sweeps from 17 to 20 kHz of 3 seconds each at a sound pressure of 70–85dB as measured at the arena floor. In some experiments overhead visual stimuli were used. These were projected onto a screen positioned 1.8m above the arena floor and consisted of a dark circle (Weber contrast = −0.98) expanding over a period of 250ms.[2] The visual stimulus was repeated five times in short sequence with an interval of 500ms between repeats. No

difference in behavior was observed between auditory and visual stimuli and therefore the data were pooled. Stimuli were triggered manually and controlled with software custom-written in LabVIEW (MANTIS, 2015 64-bit, National Instrument). While manual stimulation could be a source of bias, the arena design ensured that mice would be in the same position and similar orientation across trials and experiments. No systematic difference in position or orientation was observed based on the selected escape path. The sound was played from the computer through an amplifier (TOPAZ AM10, Cambridge Audio) and speaker (L60, Pettersson). The audio signal was fed in parallel through a breakout board (BNC-2110, National Instruments) into a multifunction I/O board (PCIe-6353, National Instruments) and sampled at 10 KHz. To synchronize the audio and video, this signal was compared to the 30/40 Hz pulse triggering video frame acquisition, which was also fed as an input to the input/output board and sampled at 10 KHz. The visual stimuli and Video S were synchronized using a light dependent resistor whose voltage output depends on the amount of light it detects and thus reflected the presence/absence of visual stimuli. The resistor's output was fed as input to the input-output board and sampled at 10 KHz.

Behavioral assay

Experimental sessions were filmed at 30 or 40fps with an overhead camera (positioned 1.8m above the arena floor). At the start of each experimental session mice were allowed to freely explore the arena for a period of ~10 minutes, during which they spontaneously found the shelter. For experiments where the configuration of the arena was reversed, we allowed mice to explore the new configuration for ~15 minutes. The additional ~5 minutes serve to compensate for the additional time the mice spend in the shelter after threat presentation and generate an amount of active exploration comparable to the baseline configuration. After the exploration period, threats (either auditory or visual) were presented repeatedly while the animals were on a designated threat platform and facing in the direction opposite from the shelter platform. A stimulus response was considered an escape if the mouse reached the shelter within 10 seconds from stimulus onset. The number of trials in each experimental session varied across mice. Experiments were terminated when mice either remained in the shelter continuously for 30 minutes or failed to escape in response to three consecutive stimuli. Some experiments were performed in total darkness (with auditory stimuli only). No difference in behavior was observed between lights on and lights off experiments and thus the two datasets were pooled.

Animal tracking

The position and orientation of mice in the arena was reconstructed using DeepLabCut[25] to track the position of several body parts (snout, neck, body and tail base) in all recorded Video Ss with a custom trained network. Post processing of tracking data included median filtering (window width: 7 frames), removal of low-confidence tracking (likelihood < 0.995) and affine transformation to a standard template arena to facilitate comparison across experiments.[3] Processed tracking data were stored in a custom DataJoint database[26] which also stored experimental metadata (e.g., mouse number, arena type, stimuli times etc.) and was used for all subsequent analysis.

Analysis code

All analysis was carried out using custom Python code and used several software packages from Python's scientific software ecosystem: NumPy,[27] Matplotlib,[28] Scikit,[29] Pandas,[30] OpenCV,[31] and StatsModels.[32] To calculate the animal's orientation, we computed the vectors between the tail base and body and between the body and snout body part location as reconstructed by DeepLabCut, we then took the average of the two (we found this to be more stable than either vector alone). We set the shelter direction to be at 0 degrees in allocentric coordinates.

Reinforcement learning modelling

Three classes of Reinforcement Learning (RL[17]) models were trained to navigate a grid world representation of the Arenas 2, 3 and 4 from the experimental study. All RL simulation, analysis and data visualization work were done in custom Python code. The grid world representation of Arena 4 consisted of a 50x50 array of quadrilateral cells with zeros corresponding to locations on the arena (126 cells in total) and ones to locations inaccessible by the agent. Agents could move in 8 cardinal directions (up, up-right, right, down-right, down, down-left, left, up-left) by one cell at the time and had to learn how to navigate the environment from a starting location to a goal location (corresponding to the threat and shelter locations in Arena 4 correspondingly). All agents were awarded a reward of 1 for reaching a cell < 3 cells distant from the goal location and received a penalty of -0.1 for attempting a move leading towards an inaccessible cell (upon which the agent did not move). For the QTable agent only (described below) a small (1×10^{-8}) reward was also delivered for any training step in which the agent moved to a new cell to encourage exploration.

Reinforcement learning models

Three different reinforcement learning model were used. All models shared the same environment (state space), actions space and reward function (with the exception of QTable as noted above). The three models were: QTable (model free RL[17]), DYNA-Q (model based[17]) and Influence Zones (IZ, model based[18]). These models were selected because they represent different classes of reinforcement learning algorithms. The QTable algorithm is a standard model-free RL algorithm. These algorithms typically require extensive amounts of training to learn simple tasks and struggle to learn tasks with large state spaces. QTable learns the value associated with taking each action at each state through temporal difference (TD) learning, which computes the difference between the expected and the experienced reward. TD is slow to update action values, requiring repeated experiences, and therefore model-free

algorithms learn slowly and cannot rapidly adapt to changes to the environment (e.g.: maze configuration). In contrast, algorithms such as DYNA-Q belong to the class of model-based algorithms, which can learn is a rapid and flexible manner. At the core of these algorithms there is a model that stores information about consequences of previously experienced actions (e.g.: rewards and state transitions). The model can then be used to update action values using TD learning in an offline mode. Because this offline learning does not require interaction con the environment, model-based algorithms such as DYNA-Q can rapidly learn how to solve a wide range of reinforcement learning problems. The IZ algorithm is also model-based but was specifically designed to efficiently solve a spatial navigation problems. The model in IZ is a simplified map of the environment that captures essential information, such as the environment's topology, but is composed of much fewer nodes than there are states of the world, which drastically reduces state space and simplifies the learning problem. IZ then uses TD to learn how to navigate this simplified map to reach the goal, and actions that move in the map can be easily translated into actions moving the agent in the corresponding state space. The map itself reflects the geometry of the environment and it is learned during exploration using a previously described algorithm called Self Organizing Map.[33] Importantly, map learning does not depend on reward and can thus proceed rapidly in the face of a sparse reward structure. In summary, these three algorithms cover simple model free learning, model based, and model based targeted to spatial problems.

All three models were implemented in custom python code. For all simulations the parameters were used are shown in Table S1. The DYNA-Q model includes a planning step in which randomly sampled entries from the agent's model are used to update the value function. In all simulations the number of samples used for each planning step was set to 20. The IZ model had additional parameters. These include a TD error threshold for one-step updates of value function (1e−10) and a threshold for n-step updates (0.1) and additional parameters for the Instantaneous Topological Map (ITM; [33]) model used by IZ: ITM learning rate (0.2) and max error (1.2).

Free exploration training

In the free exploration training regime, agents were trained for 250 episodes of maximum 500 training steps each. For each training episode the agent was initialized in a random location on the grid world arena and had to navigate to the goal location. The episode terminated when the agent took 500 steps or if it reached the goal location. During training at each step agents selected the action to perform using an epsilon greedy strategy: a random action is chosen with probability equal to the exploration rate parameter, otherwise the action with the current highest value is selected. At the end of each episode, the exploration rate decayed by a factor set by the parameter exploration rate decay.

To assess the agent's performance during learning, at the end of each training episode the agent was initialized at the start location and allowed to act greedily (i.e., with no randomly selected actions). If the agent reached the shelter location the simulation was marked successful, otherwise it was labelled as a failure. If the agent attempted an illegal move (i.e., trying to move to an inaccessible cell) the simulation was terminated and considered a failure. The agents were not allowed to use the experience from this evaluation simulation for learning.

Guided exploration training

In the guided exploration training, RL agents followed the exploration trajectory from the experimental animals. Tracking data from each experiment's exploration phase was registered to the grid world arena through an affine transformation (scaling and shift). Tracking data was represented at a higher spatial resolution that the grid world arena and did not match the grid world arena layout perfectly (due to imperfect registration of the tracking data to the standard template). The first issue was resolved by assigning, for each frame in the tracking data, the grid world arena cell closest to it. Imperfect alignment and tracking errors could not be corrected in some experimental sessions and these were discarded, leaving 42 valid sessions. As mice often remained in the same location for extended periods of time during natural exploration (e.g., in the shelter), these periods were eliminated from the tracking data and only frames in which the mouse moved from one arena cell to another were kept. The tracking data was then used to guide the movement of all agents during the training phase. For a given session's data, the agent was initialized at the arena location corresponding to the first frame in the tracking data. For each step, the location of the next arena cell was identified and the action leading from the agent's current cell to the next was identified. The agent then performed the selected action, experienced rewards and learned, as it would have during free exploration. Thus, the main difference between the free and guided exploration paradigms was that in the guided exploration regime agents were not allowed to select which action to perform as this was determined by the tracking data.

Once the agent followed each step from the tracking data the training phase was concluded. The agent was then initialized at the start location and allowed to act greedily following the value function it learned during training, with the goal of reaching the shelter location. If the goal was reached the simulation was classified as successful, otherwise it was classified as a failure. If the agent attempted an illegal move (i.e., trying to move to an inaccessible cell) the simulation was terminated and classified as a failure.

QUANTIFICATION AND STATISTICAL ANALYSIS

Quantification of escape probability

To calculate the probability of escaping in response to the threat stimulus, the movement trajectories during the first 10s after stimulus onset were analyzed, and the fraction of trajectories terminating on the shelter platform was computed. For comparison, randomly selected time points in which the same animals where on the threat platform but not presented with a threatening stimulus

were selected, and the fraction of shelter arrivals in this random sample was estimated too. The number of randomly selected time points matched the number of trials. Fisher's exact test was used to determine the significance of the difference in number of shelter-arrival trajectories between stimuli and control groups.

Quantification of the probability of escaping along a dead-end

To determine the probability of escape along the dead-end arm in arena 1, all trials from experiments in the arena were pooled. The trajectory in the 10 seconds following threat presentation was analyzed to determine which arm the mouse first moved to after leaving the threat platform, and the probability of escape along each of the three arms (left, right and dead end) was then computed. To distinguish between trials in which the mice escaped vs trials in which the mouse ignored the stimulus but still moved away from the threat platform, we used two criteria. First, since escapes are characterized by a higher running speed than normal locomotion,[2] we only included in the analysis trials in which the mouse was moving at a speed higher than 35 cm/s when it left the threat platform. Second, as escapes have fast reaction times and mice leave the threat platform within the first 3 seconds from stimulus, only trials in which the mouse left the threat platform within 4 seconds from stimulus onset were considered escapes. The same analysis was performed on all trials from the open-arm configuration of arena 1 (with the central path leading to the shelter), and the probability of escape along the central arm estimated for both datasets. The significance of the difference in escape probability between arena configurations was determined with a two proportions z-test.

Quantification of heading direction

To quantify the average heading direction during escape, movement trajectories from each arena were selected and grouped into left or right path escapes. The trajectories where then truncated to the frame in which the mouse left the threat platform and their duration normalized. The average heading direction at regular intervals across all trajectories in the same group was then computed.

Quantification of arm choice probability

To estimate the probability of escape along a given path, all trials from experiments on each arena were pooled and the probability of selecting the right or left arm was computed. To assess the effect of arena design on arm choice probability, a χ^2 test was used to test whether the probability of escaping on the right vs left path was significantly different from chance for asymmetric arenas.

In experiments in which the arena design changed during the course of an experiment, trials were pooled across animals and grouped into baseline (before the change) and 'flipped' (after the change). Fisher's exact test was used to determine whether the number of escapes along the right path differed between the baseline and flipped conditions.

In addition to estimating the probability of selecting an arm, the posterior distribution of the probability value was estimated using a Bayesian model. The model had a Beta distribution prior (parameters: a=1, b=1) and a Binomial distribution as likelihood (n = total number of trials, k = total number of right path escapes). The resulting posterior distribution is then a Beta distribution whose parameters are given by:

$$a_{posterior} = a_{prior} + k - 1$$

$$b_{posterior} = b_{prior} + n - k - 1$$

To compare the probability of selecting the right arm between naïve and experienced trials, for each arena the first escape trial for each mouse was classified as naïve while all other trials were classified as experienced. When a mouse was used for more than one experiment, only the first trial on the first experiment the mouse was used for was considered naïve. The probability of escaping along the right path was computed for the naïve trials. Because the number of experienced trials is larger than the naïve condition, to compare the probability of taking the right path between the two groups, experienced trials were randomly sampled without replacement to match the number of naïve trials in the same arena, and the probability of taking the right path was then computed. This procedure was repeated 10 times to generate a distribution of probability values, and the mean and 95th percentile interval were computed from this distribution.

Arm choice probability for naïve vs experienced trials

To determine whether mice required repeated experience with threat to select the preferred escape path we identified the very first stimulus presentation of each animal. We grouped such "naïve" trials across individuals tested on the same experimental arena and estimated the probability of escape along the right path in this subset of the data. To compare naïve vs experienced (i.e. following the first encounter with threat) trials, we randomly sampled from the experienced trials from each arena matching the number of naïve trials in the same arena and we computed the probability of escape on the right arm. We repeated the sampling procedure 100 times to obtain a distribution of probability values for different random subsets.

Change in path preference with time

To quantify the change in probability of escape along the right path over time, for each arena we pooled all trials that occurred during the first 60 minutes from the start of the experiment. We then binned the trials based on the time since experiment start (interval between bins: 120 seconds, bin width: 300s) and computed the posterior distribution of p(R) as described above.

Quantification of shelter-threat trips during exploration

To quantify the number of trips between the shelter and the threat platform during exploration, the tracking trajectory corresponding to the exploration period (from start of the experiment to one frame before the first stimulus) was analyzed. For each frame, the mouse was assigned to one of four regions of interest ('shelter', 'right arm', 'threat', 'left arm') based on the coordinates of the tracking data registered to a standard template image as described above. A trip was ended when the mouse arrived at the shelter (threat) platform and started at the last frame in which the mouse was on the threat (shelter) platform. Incomplete trips (e.g.: the mouse left the shelter platform and returned to it without first reaching the threat platform) were discarded.

Predicting escape arm with GLM

To predict the probability of escaping along the right arm from trial data, a binomial generalized linear model (GLM) with a logit link faction was used (implemented in StatsModels; [32]). All trials from all arenas were pooled and split between train and test sets (stratified k-fold repeated cross-validation; five different splits of the trials were used, and the data were split such that the test set was roughly balanced with respect to the number of trials from the corresponding arena; this procedure was repeated four times with different random splits each time, yielding a total of 20 model fits). The GLM model attempted to estimate the probability of escape along the right path for each trial based on: 1) the geodesic ratio of the trial's arena, 2) the angles ratio of the trial's arena, 3) the trial time (in seconds) since the start of the experimental session, 4) the identity of the origin arm. Categorical variables were one-hot encoded, and all variables were normalized to the 0–1 range. The accuracy of the model's predictions on the test data was estimated with the Pearson's correlation between the predicted probability of escape on the right arm and the arm chosen by the animal. This accuracy measure was compared to the accuracy of models fitted on randomly shuffled data. The full model described above was then compared to models lacking one or two of the input variables to estimate the effect of each variable. Each model was fitted on k-fold cross validated data (k=5) and the procedure was repeated four times using different random number generator seeds for each repeat. The coefficient weights of each parameter for each fit of the full model were used to estimate the average and standard deviation of the coefficient weights.

Decoding escape path from threat trajectory

To decode the escape arm from the trajectory on the threat platform we used a logistic regression model (implemented in Scikit; [29]). Trajectories from all trials in each experimental arena were pooled and their duration was normalized. To assess how the model performed as mice moved away from the threat location and towards the escape arms, 8–9 time points were selected corresponding to different average positions along the axis between the threat and shelter platform. For each time point, the animal's average orientation in the five frames after the time point and the trial's escape arm were used. The data were randomly split between a training and test set (test set 0.33% of the trials) and the training set was used to fit the model to predict the escape arm based on the orientation value. The model's accuracy score on held out training data was then computed. The procedure was repeated 100 times for each time point with a different random split of the data and the average accuracy computed.

Quantification of RL models performance

To quantify the performance of RL agents trained to navigate the grid world arena under the free exploration regime, we trained 64 repetitions of each model. At the end of each training episode, each repetition was tested on its ability to navigate to the goal location and returned a 1 for successes and 0 otherwise. Thus, a vector of outcomes was constructed based on the value returned by each repetition and the overall score was given by the mean and standard error of the mean (SEM) of the outcomes vector. For visualization, the mean and SEM accuracy at each training episodes were displayed following smoothing with a rolling mean filter with window width of 6 episodes.

To quantify the performance of RL agents trained under the guided exploration regime, we trained 10 repetitions of each RL model on the tracking data from each experimental session. Under this regime, unlike in free exploration, training is fully deterministic because the actions are specified by the tracking data, the only variability emerges from the DYNA-Q model's probabilistic sampling of its model at each training step. After training, all repetitions of each model were tested on their ability to navigate from the start to the threat location. The model was considered successful if at least 8/10 repetitions successfully reached the goal location.

To produce the state occupancy heatmaps in Figure 3B we recorded all cell visits for one example agent trained under the free exploration regime and one example agent trained under the guided exploration regime. The total number of visits to visits to each cell was then used to produce the heatmap. To visualize the preferred action at each cell (Figure 3E) we trained one example agent for each class of RL algorithm and training regime and displayed the action with highest value for each cell.

Current Biology, Volume *32*

Supplemental Information

Innate heuristics and fast learning support escape route selection in mice

Federico Claudi, Dario Campagner, and Tiago Branco

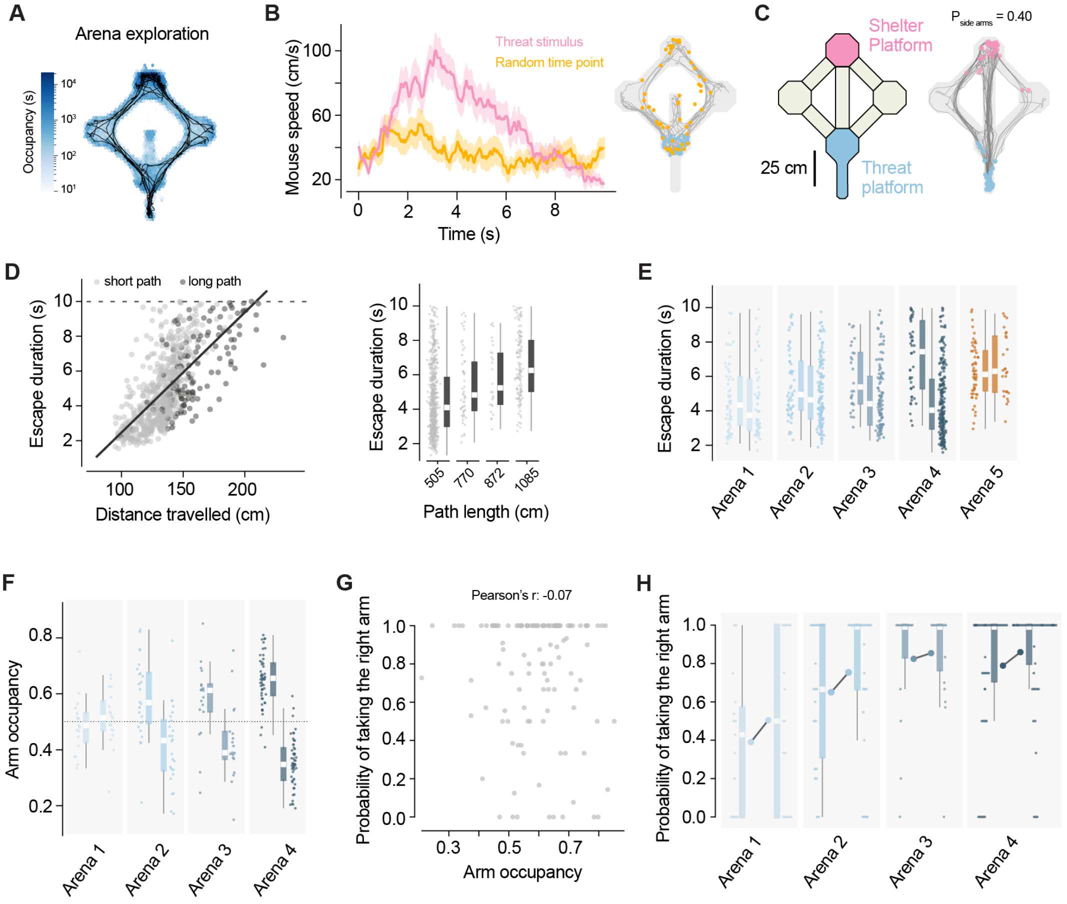

Figure S1 - Escape duration correlates with distance, and route choice does not depend on path exploration, Related to Figure 1.

(A) Heatmap of arena occupancy during exploration of Arena 1 with example tracking traces from one mouse (black). **(B)** Left, speed trajectory in the 10s following the threat stimulus (pink) or a randomly selected time point when the mouse was in the threat area (yellow). Probability of reaching the shelter after threat stimulus = 0.65 vs 0.17 for random time points (p = 0.00097, Fisher exact test). Bold curves and shaded areas show mean and standard deviation across trials. Right, tracking traces for the random time point condition, blue circles show the starting location, orange circles the final location. **(C)** Left, schematic view of a version of Arena 1 with the central arm open. Right, movement tracking traces for all escape trials (grey) for Arena 1 with the central arm open (c.f. Figure 1A). Blue and pink circles mark the start and end of each escape run, respectively. **(D)** Left, correlation between distance travelled during escape and escape duration. Each dot represents a single escape trial, darker dots are trials in which the animals selected the longer paths (in Arenas 2,3,4). Black line shows best fit of linear regression (slope: 0.07, intercept: -4.4). Right, escape duration along paths of different lengths. Each dot represents a single trial, box plots show the median and 95th percentile of the distribution. **(E)** Escape duration for left and right path escapes across arenas (for each arena the left bar shows escapes on the left and the right bar shows escapes on the right). Each dot represents a single trial, box plots show the median and 95th percentile of the distribution (t-test with Bonferroni correction for comparison between left and right: Arena 1, p=1.0; Arena 2, p=0.5; Arena 3, p=0.04; Arena 4, p=1.9e-12; Arena 5, p=1.0). **(F)** Fraction of time spent on left and right path during exploration normalized by the total time spent exploring (shown by bars on the left and right for each arena, respectively; dashed line marks equal occupancy for both arms). Circles indicate individual mice, box plots show the median and 95th percentile of the distribution (t-test with Bonferroni correction for comparison between left and right: Arena 1, p=1.0; Arena 2, p=0.0003; Arena 3, p=0.0066; Arena 4, p=8.2e-32;). **(G)** Plot showing that arm occupancy (calculated as in panel F) and probability of escaping through the right arm are not correlated. **(H)** Probability of escape on the right path for subsets of trials in which mice reached the threat platform from the left or the right path (shown by bars on the left and right for each arena, respectively). Colored circles represent individual mice, box plots show the median and 95th percentile of the distribution (values between left and right are not significantly different for all arenas).

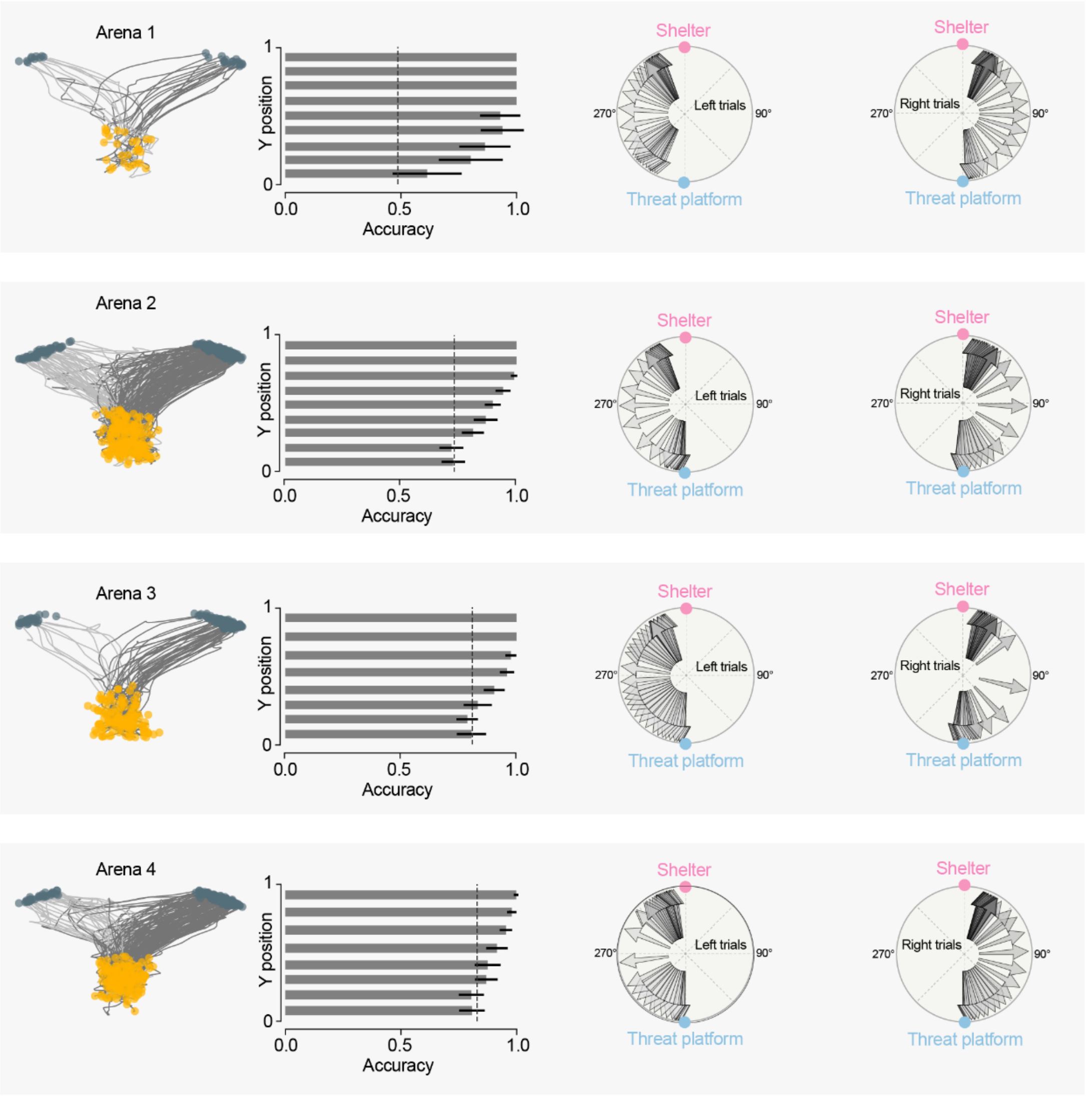

Figure S2 - Escape path choice can be decoded from the movement trajectory in the threat platform, Related to Figure 1.

Decoding of escape path selection probability based on tracking data on the threat platform. Left, tracking traces for all trials in each arena. Darker traces show escapes on the right path, orange circles the initial position and blue circles the animal's position at the time of leaving the threat platform. Center, test set accuracy of a logistic model predicting escape path choice based on tracking data binned along the threat-shelter axis. Right, average orientation across trials during escape progression for left and right trials. Arrows show orientation for each time bin, darker colors indicated later time points.

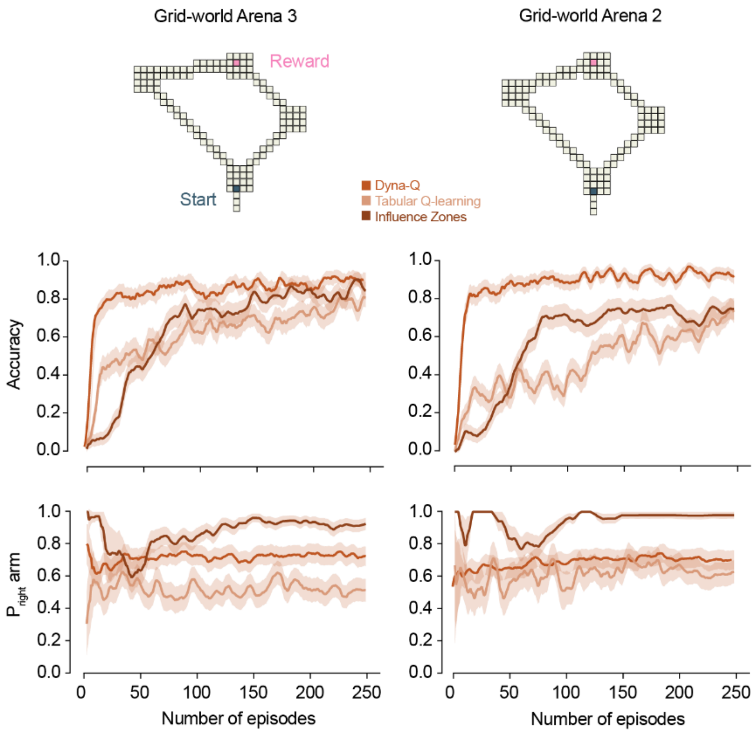

Figure S3 - Model performance in Arenas 2 and 3, Related to Figure 3.

Top row, schematics of the grid world arenas used for simulating Arenas 2 and 3. Middle row, evolution of accuracy during learning for the three different RL models under ε-greedy exploration. Bottom row, probability of choosing the right arm in successful trials during training. Note that for Dyna-Q, the probability of choosing the right arm in Arena 2 is slightly lower than in Arena 3, and both probabilities are lower than for Arena 4 (c.f. Figure 3D), which qualitatively matches the experimental data.

	DISCOUNT	LEARNING RATE	EXPLORATION RATE	EXPLORATION RATE DECAY
**QTABLE**	0.8	0.9	0.4	0.995
**DYNA-Q**	0.8	0.8	0.4	0.995
**INFLUENCE ZONES**	0.8	0.2	0.4	0.995

Table S1 – Parameters for the reinforcement learning agents, Related to Figure 3.

Parameter values for the reinforcement learning agents.
